# Genetic and Clinical Determinants of Variation in Drug Response in Type 2 Diabetes: Insights From the Scottish and UK Biobank Cohorts

**DOI:** 10.1111/dom.71262

**Published:** 2026-09-01

**Authors:** S. Garg, R. Kitchen, R. Gupta, L. Donnelly, E. R. Pearson

**Affiliations:** ^1^ Diabetes Endocrinology and Reproductive Biology University of Dundee Dundee UK; ^2^ Novo Nordisk Research Centre Oxford Oxford UK

**Keywords:** beta cell function, cohort study, glycaemic control, pharmacogenetics, type 2 diabetes

## Abstract

**Objective:**

Treatment response in type 2 diabetes (T2D) varies widely among individuals. This study aimed to quantify the contributions of clinical characteristics and genetic predisposition as measured through partitioned polygenic risk scores (pPRS) to variation in glycemic response to glucose‐lowering therapies.

**Research Design and Method:**

We analysed data from two population‐based cohorts: the Genetics of Diabetes Audit and Research in Tayside Scotland (GoDARTS) and the UK Biobank (UKBB). GoDARTS included 41 802 patients who initiated one of six major drug classes, of whom 11 615 had available genotype data. UKBB contributed 9371 individuals, including 8293 with genetic data. The primary outcome was glycaemic response, defined as the change in HbA1c 12 months after treatment initiation. Variables included demographic and clinical factors (age, sex, BMI, baseline HbA1c, kidney and liver function markers) and 14 pPRS representing T2D‐related biological pathways. Linear regression models were fitted within each cohort and drug class (metformin, sulfonylureas, TZDs, DPP4i, SGLT2i, GLP‐1RA), and effect estimates were combined using fixed‐effect meta‐analysis.

**Result:**

Baseline HbA1c was most strongly associated with glycemic response (*p* < 0.001). Older age was consistently associated with greater HbA1c reduction, while BMI and total cholesterol demonstrated drug‐class‐specific associations, with higher BMI associated with improved response to TZDs and higher total cholesterol generally associated with poorer glycaemic outcomes. Meta‐analysis across GoDARTS and UK Biobank showed that higher overall T2D genetic risk was associated with greater HbA1c reduction with sulfonylureas (*β* = −0.46 mmol/mol, *p* = 0.013). Specific genetic profiles were also associated with drug responses, including β‐cell function clusters with sulfonylureas (*β* = −0.57, *p* = 0.002), obesity‐related variants with GLP‐1RA (*β* = −1.49, *p* = 0.04), liver‐lipid variants with SGLT2 inhibitors (*β* = −0.84, *p* = 0.05), and bilirubin pPRS with DPP‐4 inhibitors (*β* = −0.69, *p* = 0.006).

**Conclusion:**

Both clinical and genetic factors significantly contribute to inter‐individual variability in T2D drug response. Partitioned PRSs provide mechanistic insights into drug‐specific pathways and have the potential to inform precision prescribing and optimise therapeutic outcomes in routine diabetes care.

**Abbreviations**
ALP: alkaline phosphatase
ALT/SGPT: alanine aminotransferase/serum glutamate pyruvate transaminase
BMI: body mass index
eGFR: estimated glomerular filtration rate
EHR: electronic health records
GRS: genetic risk score
HbA1c: glycated haemoglobin
HDL: high‐density lipoprotein
MICE: multivariate imputation by chained equations
pPRS: partitioned polygenic risk score
R^2^
: R‐squared (coefficient of determination)
SBP: systolic blood pressure
SU: sulphonylurea
T2DM: type 2 diabetes mellitus
TZDs: thiazolidinediones
VIF: variance inflation factor

## 1 Introduction

Treatment response in type 2 diabetes (T2D) is highly heterogeneous, with substantial variability in glycaemic outcomes even when patients receive the same glucose‐lowering therapies [1]. Such variability complicates treatment selection, often resulting in a trial‐and‐error prescribing approach that can delay optimal glycaemic control and increase the risk of diabetes‐related complications [2]. Understanding the determinants of inter‐individual differences in drug response is essential for advancing precision medicine in T2D.

Clinical characteristics such as age, sex, BMI, renal function and baseline HbA1c are well‐established variables associated with glycemic response [3, 4], but they explain only part of the observed variation. Genetic predisposition is increasingly recognized as another contributor to inter‐individual differences in drug efficacy, metabolism and tolerability [5]. From a pharmacogenomics (PGx) perspective, genetic factors can influence treatment outcomes through disease aetiology and underlying metabolic pathways, as well as through pharmacokinetic and pharmacodynamic mechanisms [6]. While pharmacokinetic variants can exert large functional effects, they are relatively rare; in contrast, common variants influencing disease processes may affect treatment response by modulating insulin secretion, insulin resistance, or metabolic regulation [7, 8, 9, 10].

Polygenic risk scores (PRS) provide a quantitative measure of an individual's inherited susceptibility by aggregating many common risk alleles identified through genome‐wide association studies [11]. More recently, pathway‐partitioned PRS (pPRS) have been developed to improve biological interpretability by grouping variants into mechanistically coherent clusters. These scores have been successfully applied to investigate disease heterogeneity and related clinical traits in type 2 diabetes [12]. However, their application to treatment response has not been comprehensively evaluated in large real‐world cohorts. In T2D, frameworks proposed by Smith et al. [13] and Suzuki et al. [12] have defined clusters reflecting heterogeneity in β‐cell function, obesity‐related insulin resistance, liver–lipid metabolism and general metabolic regulation.

Major glucose‐lowering drug classes target many of the same physiological pathways implicated in type 2 diabetes pathogenesis. Metformin enhances hepatic insulin sensitivity; sulfonylureas stimulate pancreatic β‐cell insulin secretion; thiazolidinediones activate PPAR‐γ to improve peripheral insulin sensitivity; and DPP‐4 inhibitors, GLP‐1 receptor agonists and SGLT2 inhibitors modulate incretin biology and renal glucose handling [14, 15]. Variability in these physiological mechanisms may interact with genetic background, thereby shaping the glycemic response to therapy [16]. Given this alignment between drug mechanisms and genetically defined metabolic pathways, pathway‐specific polygenic risk scores (PRS) may help explain differential glycaemic responses by linking an individual's genetic architecture directly to the physiological pathways targeted by therapy [17, 18].

Although previous research has established the value of routinely collected clinical predictors for treatment response, including recent clinical prediction models for drug‐class selection [4], and other studies have examined selected genetic predictors associated with response to individual drug classes, no prior study has comprehensively evaluated the combined contribution of clinical characteristics and partitioned polygenic risk scores across all major glucose‐lowering therapies in large real‐world cohorts. In this study, we assess the association of both clinical features and partitioned PRS with glycaemic response across six major glucose‐lowering drug classes, using data from two large and well‐characterised cohorts: GoDARTS and the UK Biobank. By integrating clinical and genetic information, we aim to improve understanding of the biological determinants of drug response and to inform strategies for more personalized diabetes management.

## 2 Research Design and Methods

### 2.1 Cohorts

This retrospective study utilized electronic health records (EHRs) and genetic data from two well‐characterised resources: GoDARTS (Genetics of Diabetes Audit and Research in Tayside Scotland), comprising individuals from the Tayside and Fife regions of Scotland [19], and the UK Biobank (UKBB), a large prospective population‐based cohort [20]. The GoDARTS dataset includes records spanning 1995–2025, while the UK Biobank dataset covers 2006–2017. Both cohorts provided detailed prescribing information, laboratory measurements, demographics, genome‐wide genotyping and longitudinal follow‐up data, enabling a comprehensive assessment of clinical and genetic variables associated with treatment response.

### 2.2 Study Design

We included individuals with type 2 diabetes who initiated therapy with one of six major glucose‐lowering drug classes: metformin, sulfonylureas (SU), DPP‐4 inhibitors, TZDs, SGLT2 inhibitors and GLP‐1 receptor agonists. Eligible patients had no prior exposure to the same drug within the 12 months before treatment initiation. Continuous prescriptions were required to ensure sufficient follow‐up after initiation. Patients were categorised as receiving monotherapy (first‐ever drug), dual therapy (drug added to one existing agent), or triple therapy (drug added to two existing agents).

### 2.3 Assessment of Glycemic Response

The primary outcome was change in HbA1c from baseline to approximately 12 months after treatment initiation. Baseline HbA1c was defined as the measurement closest to the initiation date, within a window of −183 to +7 days and restricted to values between 53 and 140 mmol/mol (7%–14%) to ensure clinical relevance. Follow‐up HbA1c was defined as the measurement obtained between 6 and 15 months after initiation that was closest to 12 months. If multiple measurements were available within this window, the value nearest to 12 months was selected. Only one baseline and one follow‐up HbA1c measurement were included per individual.

### 2.4 Baseline Clinical Variables

Clinical covariates included age, sex, BMI, diabetes duration, renal function (serum creatinine, eGFR), liver function (ALT, alkaline phosphatase, bilirubin), metabolic markers (total cholesterol, HDL cholesterol, albumin, potassium), smoking status, systolic blood pressure. These variables were selected based on their availability in routine care and their known or suspected relevance to glycaemic outcomes [21]. Except for baseline HbA1c, all measurements were defined as the closest recorded value within 2 years before to 90 days after drug initiation. Average daily drug dose was standardised and categorised into low, medium and high using tertiles.

Definitions of clinical variables used in the analysis are provided in Table S1.

### 2.5 Genetic Variables

Genetic predisposition was assessed using partitioned polygenic risk scores (pPRS). Fourteen European‐ancestry pPRS were derived from effect sizes reported by Smith et al. [13], capturing biological mechanisms such as beta‐cell function, insulin resistance, obesity, lipid metabolism and liver‐related pathways. Each pPRS was analysed separately in regression models of HbA1c response, adjusted for relevant demographic, anthropometric and clinical covariates, in both GoDARTS and UKBB.

### 2.6 Data Preparation

Continuous variables with skewed distributions were log‐transformed [22], enhancing model stability and reducing the influence of extreme values. Categorical variables (e.g., sex, smoking status, treatment group, average daily dose) were converted using one‐hot encoding. Missing data were imputed using multiple imputation by chained equations (MICE) [23]. This approach generates multiple complete datasets by imputing missing values for each variable using regression models conditional on other observed variables in the dataset. Multicollinearity was assessed using variance inflation factors (VIF) [24]. As eGFR and creatinine were strongly correlated, only creatinine was retained due to its routine clinical use and age and sex were used independently in the models.

### 2.7 Statistical Analysis

#### 2.7.1 Clinical Models

We first tested associations between baseline clinical variables and HbA1c response using multivariable linear regression for each drug class. Models included demographic, anthropometric (age, sex, duration of diabetes), and above‐mentioned all clinical variables (Table S2). Heatmaps were used to summarise drug‐class‐specific clinical variables.

#### 2.7.2 Genetic Models

Genetic variables were evaluated by including each pPRS individually in linear regression models, adjusted for demographic, anthropometric and a subset of clinical covariates (Table S2). Analyses were conducted separately in GoDARTS and UKBB and results were combined using fixed‐effect inverse‐variance meta‐analysis [25]. Forest plots illustrated associations between pPRS and treatment response.

#### 2.7.3 Multiple Testing Correction

To account for multiple statistical comparisons, Benjamini‐Hochberg False Discovery Rate (FDR) correction was applied to the fully adjusted (Model 3) meta‐analysis results separately for each drug class. Within each drug class, the 15 genetic association tests (14 partitioned polygenic risk scores and one total genetic risk score) were considered as a single family of hypotheses. Both nominal *p*‐values and FDR‐adjusted q‐values are reported in Table S10. Associations with *q* < 0.05 were considered statistically significant after correction.

#### 2.7.4 Variance Explained by Clinical and Genetic Variables

To assess the relative contribution of individual variables, we calculated partial *R* ^2^ values. Partial *R* ^2^ was defined as the proportion of variance in drug response uniquely explained by a given variable after adjusting for all other covariates in the model [26]. In this analysis, partial *R* ^2^ estimates are reported alongside regression coefficients (*β*) and *p*‐values in the Tables S4–S9.

All statistical analyses were performed using R (version 4.3.0). The packages used throughout the analyses are listed in Table S3.

## 3 Results

### 3.1 Cohort Characteristics

In GoDARTS, 41 802 patients initiated one of six drug classes, while in UK Biobank (UKBB), 9371 patients did so. Baseline characteristics were broadly similar across cohorts (Table 1). Not all patients had genotype data available: in GoDARTS, 11 615 patients were genotyped, and in UKBB, 8293 of 9371 patients had genotype data. Data are presented as mean (SD) or *n* (%), where *n* represents the number of patients who initiated each drug class during follow‐up.

**TABLE 1:** Baseline characteristics of patients.

			GoDARTs					
	
	Metformin (*n* = 19 801)	SU (*n* = 8276)	DPP4 (*n* = 5983)	TZD (*n* = 2918)	SGLT2 (*n* = 4065)	GLP1 (*n* = 759)
1		Age at therapy initiation (years)	62.9 (12.3)	63.9 (11.8)	64.1 (11.7)	62.2 (10.8)	61.9 (11.0)	58.8 (10.7)
2		Sex (*N* [%])						
		Male	11 500 (58.1%)	4786 (57.8%)	3597 (60.1%)	1727 (59.2%)	2531 (62.3%)	460 (60.6%)
		Female	8301 (41.9%)	3490 (42.2%)	2386 (39.9%)	1191 (40.8%)	1534 (37.7%)	299 (39.4%)
3		Average daily dose (*N* [%])						
		Low	6601 (33.3%)	2759 (33.3%)	1995 (33.3%)	973 (33.3%)	1355 (33.3%)	253 (33.3%)
		Medium	6600 (33.3%)	2759 (33.3%)	1994 (33.3%)	973 (33.3%)	1355 (33.3%)	253 (33.3%)
		High	6600 (33.3%)	2758 (33.3%)	1994 (33.3%)	972 (33.3%)	1355 (33.3%)	253 (33.3%)
4		Duration of diabetes (years)	3.01 (3.9)	5.3 (4.6)	8.16 (5.46)	7.3 (5.0)	9.1 (5.6)	9.9 (5.7)
5		Time of treatment (months)	11.3 (2.3)	11.4 (2.25)	11.0 (2.43)	11.3 (2.2)	10.8 (2.5)	10.6 (2.6)
6		Time to treatment start (days)	18.9 (26.1)	21.5 (29.2)	22.9 (28.1)	21.7 (27.7)	25.1 (29.2)	28.7 (31.5)
7		Year of drug start	2010 (6.53)	2010 (7.01)	2020 (3.83)	2010 (4.9)	2020 (2.7)	2020 (5.33)
8		BMI (kg/m^2^)	33.1 (6.67)	31.55 (6.3)	33.0 (6.59)	32.5 (6.2)	33.8 (6.7)	37.5 (7.78)
9		Total cholesterol (mmol/L)	4.78 (1.19)	4.5 (1.2)	4.14 (1.00)	4.3 (0.9)	4.2 (1.1)	4.2 (1.1)
10		HDL cholesterol (mmol/L)	1.16 (0.301)	1.2 (0.3)	1.1 (0.3)	1.2 (0.3)	1.1 (0.3)	1.1 (0.3)
11		Serum creatinine (umol/L)	77.0 (19.6)	79.6 (25.1)	80.0 (25.2)	81.8 (23.6)	73.8 (21.4)	74.7 (21.4)
12		Albumin (g/L)	41.9 (3.94)	41.9 (4.14)	41.7 (4.1)	43.6 (3.1)	40.6 (4.2)	41.0 (4.13)
13		Bilirubin (umol/L)	10.7 (5.4)	10.0 (6.53)	9.5 (4.9)	9.6 (5.1)	9.6 (4.7)	9.3 (4.9)
14		Alkaline phosphatase (U/L)	92.1 (31.3)	90.0 (36.0)	87.8 (31.5)	82.5 (27.5)	88.5 (28.2)	89.9 (31.8)
15		ALT/SGPT (U/L)	36.6 (22.7)	34.5 (22.6)	33.3 (20.2)	32.7 (18.8)	35.7 (21.3)	34.3 (20.5)
16		Potassium (mmol/L)	4.37 (0.408)	4.4 (0.4)	4.5 (0.4)	4.5 (0.4)	4.5 (0.4)	4.5 (0.4)
17		Systolic BP	137 (16.7)	137 (17.3)	135 (15.1)	137 (15.9)	135 (14.8)	137 (16.2)
18		Smoking status (N [%])						
		Ever smoked—Yes	14 074 (71.1%)	6112 (73.9%)	4257 (71.2%)	2116 (72.5%)	2757 (67.8%)	526 (69.3%)
		Ever smoked—No	5597 (28.3%)	2094 (25.3%)	1682 (28.1%)	785 (26.9%)	1279 (31.5%)	221 (29.1%)
		Unknown	130 (0.7%)	70 (0.9%)	44 (0.7%)	17 (0.6%)	29 (0.7%)	12 (1.6%)
19		Therapy group (*N* [%])						
		Mono	17 767 (89.7%)	2644 (31.9%)	262 (4.4%)	58 (2.0%)	171 (4.2%)	0
		Dual	2034 (10.3%)	4559 (55.1%)	3490 (58.3%)	1574 (53.9%)	1944 (47.8%)	141 (18.6%)
		Triple	0	1073 (13.0%)	2231 (37.3%)	1286 (44.1%)	1506 (37.0%)	374 (49.3%)
		Quadruple	0	0	0	0	444 (10.9%)	244 (32.1%)
20		Ethnicity (*N* [%])						
		White	13 719 (69.3%)	6121 (74.0%)	4751 (79.4%)	2348 (80.5%)	2844 (70.0%)	577 (76.0%)
		Others/mixed	655 (3.3%)	296 (3.6%)	251 (4.2%)	118 (4.0%)	159 (3.9%)	27 (3.6%)
		Missing	5427 (27.4%)	1859 (22.5%)	981 (16.4%)	452 (15.5%)	1062 (26.1%)	155 (20.4%)
21		Zone (*N* [%])						
		Tayside	13 675 (69.1%)	6430 (77.7%)	2920 (48.8%)	1770 (60.7%)	2559 (63.0%)	461 (60.7%)
		Fife	6126 (30.9%)	1846 (22.3%)	3063 (51.2%)	1148 (39.3%)	1506 (37.0%)	298 (39.3%)
						
22		Baseline HbA1c (mmol/mol)	73.7 (16.6)	77.5 (17.3)	73.9 (13.3)	74.1 (13.6)	76.6 (14.2)	80.1 (15.3)
23		Treatment HbA1c (mmol/mol)	58.8 (14.5)	61.5 (15.5)	66.1 (15.6)	62.5 (15.0)	64.7 (14.4)	66.0 (16.3)
24		HbA1c response	−14.9 (19)	−16.0 (18.7)	−7.8 (15.0)	−11.6 (14.8)	−11.9 (15.1)	−14.0 (17.3)
		Drug name	METFORMIN HYDROCHLORIDE	GLIBENCLAMIDE—66 (0.8%)	ALOGLIPTIN—1701 (28.4%)	PIOGLITAZONE—2055 (70.4%)	CANAGLIFLOZIN—34 (0.8%)	EXENATIDE—27 (3.6%)
				GLICLAZIDE—6605 (79.8%)	LINAGLIPTIN—43 (0.7%)	ROSIGLITAZONE—863 (29.6%)	DAPAGLIFLOZIN—718 (17.7%)	EXENATIDE MR—108 (14.2%)
				GLICLAZIDE MR—142 (1.7%)	SAXAGLIPTIN—595 (9.9%)		EMPAGLIFLOZIN—3313 (81.5%)	LIRAGLUTIDE—261 (34.4%)
				GLIMEPIRIDE −168 (2.0%)	SITAGLIPTIN—3618 (60.5%)			LIXISENATIDE—22 (2.9%)
				GLIPIZIDE—1293 (15.6%)	VILDAGLIPTIN—26 (0.4%)			DULAGLUTIDE—84 (11.1%)
				TOLBUTAMIDE—< 5 (0.0%)				SEMAGLUTIDE—257 (33.9%)
		# Patients (genetic data)	Metformin (*n* = 5066)	SU (*n* = 2959)	DPP4 (*n* = 1428)	TZD (*n* = 1141)	SGLT2 (*n* = 810)	GLP1 (*n* = 211)

		UKBB					
		Metformin (*n* = 4800)	SU (*n* = 2291)	DPP4 (*n* = 1037)	TZD (*n* = 955)	SGLT2 (*n* = 148)	GLP1 (*n* = 140)
1	Age at therapy initiation (years)	59.9 (7.6)	59.9 (7.7)	62.2 (7.6)	58.5 (7.0)	62.3 (7.2)	60.2 (7.3)
2	Sex (*N* [%])						
	Male	2992 (62.3)	1500 (65.5)	668 (64.4)	640 (67.0)	96 (64.9)	76 (54.3)
	Female	1808 (37.7)	791 (34.5)	369 (35.6)	315 (33)	52 (35.1)	64 (45.7)
3	Average daily dose (*N* [%])						
	Low	1599 (33.3)	766 (33.4)	347 (33.5)	320 (33.5)	49 (33.1)	47 (33.6)
	Medium	1600 (33.3)	761 (33.2)	344 (33.2)	318 (33.3)	50 (33.8)	46 (32.9)
	High	1601 (33.3)	764 (33.3)	346 (33.3)	317 (33.2)	49 (33.1)	47 (33.6)
4	Duration of diabetes (years)	11.7 (6.1)	11.5 (5.9)	13.9 (6.1)	10.4 (5.0)	14.6 (7.1)	13.8 (5.8)
5	Time of treatment (months)	11.4 (2.2)	11.4 (2.2)	11.0 (2.3)	11.3 (2.3)	10.4 (2.5)	10.7 (2.3)
6	Time to treatment start (days)	18.2 (23.8)	20.4 (25.9)	23 (26.9)	23.6 (29.4)	26.7 (30.8)	36.6 (34.9)
7	Year of drug start	2009 (4.0)	2009 (4.1)	2012 (2.1)	2007 (3.0)	2014 (0.7)	2012 (1.9)
8	BMI (kg/m^2^)	31.8 (5.9)	31.4 (5.8)	31.8 (5.5)	31.8 (5.8)	32.5 (5.4)	37.2 (6.4)
9	Total cholesterol (mmol/L)	4.8 (1.2)	4.4 (1.1)	4.1 (0.9)	4.2 (0.9)	4.03 (0.96)	4.03 (0.95)
10	HDL cholesterol (mmol/L)	1.1 (0.3)	1.2 (0.3)	1.1 (0.3)	1.1 (0.3)	1.1 (0.3)	1.1 (0.3)
11	Serum creatinine (umol/L)	83.5 (17.1)	83.3 (19.7)	83.1 (23.5)	85.1 (18.6)	76.6 (17.3)	78.6 (19.3)
12	Albumin (g/L)	42 (3.6)	42.2 (3.8)	42.1 (3.8)	42.2 (3.5)	41.6 (3.8)	40.5 (3.9)
13	Bilirubin (umol/L)	11.6 (6.1)	10.9 (6.8)	10.3 (4.9)	10.7 (4.9)	10.2 (5.8)	9.5 (5.8)
14	Alkaline phosphatase (U/L)	102.1 (55.3)	93.9 (51.0)	95.7 (51.4)	96.5 (54.2)	97.7 (57.8)	88.9 (35.2)
15	ALT/SGPT (U/L)	37.9 (23.2)	37.2 (24.9)	33.7 (18.7)	37.4 (19.5)	38.1 (37.9)	34.1 (17.2)
16	Potassium (mmol/L)	4.4 (0.4)	4.4 (0.4)	4.5 (0.4)	4.5 (0.4)	4.5 (0.4)	4.5 (0.4)
17	Systolic BP	134.9 (14.9)	132.8 (14.5)	132.7 (14.0)	134.8 (14.3)	132.4 (13.1)	133.8 (13.8)
18	Smoking status (N [%])						
	Ever smoked—Yes	3079 (64.1)	1490 (65.0)	667 (64.3)	612 (64.1)	94 (63.5)	93 (66.4)
	Ever smoked—No	1721 (35.9)	801 (35.0)	370 (35.7)	343 (35.9)	54 (36.5)	47 (33.6)
	Unknown	0	0	0	0	0	0
19	Therapy group (*N* [%])						
	Mono	4396 (91.6)	355 (15.5)	0	0	0	0
	Dual	404 (8.4)	1728 (75.4)	434 (41.9)	438 (45.9)	41 (27.7)	24 (17.1)
	Triple	0	208 (9.1)	603 (58.1)	517 (54.1)	63 (42.6)	84 (60.0)
	Quadruple	0	0	0	0	44 (29.7)	32 (22.9)
20	Ethnicity (*N* [%])						
	White	4217 (88.1)	2039 (89.4)	881 (85.2)	845 (88.7)	131 (88.5)	124 (89.2)
	Others/mixed	535 (11.2)	230 (10.1)	145 (14.0)	103 (10.8)	16 (10.8)	13 (9.4)
	Missing	33 (0.7)	12 (0.5)	8 (0.8)	5 (0.5)	1 (0.7)	2 (1.4)
21	Zone (*N* [%])						
	Tayside	4442 (92.5)	2120 (92.5)	938 (90.5)	900 (94.2)	112 (75.7)	131 (93.6)
	Fife	353 (7.4)	171 (7.5)	97 (9.4)	55 (5.8)	36 (24.3)	9 (6.4)
		5 (0.1)	0	2 (0.2)	0	0	0
22	Baseline HbA1c (mmol/mol)	69.6 (15.8)	72.0 (15.1)	71.7 (12.9)	71.5 (12.5)	76.3 (15.6)	77.7 (15.3)
23	Treatment HbA1c (mmol/mol)	56.2 (12.2)	58.8 (13.9)	63.9 (14.9)	60.9 (14.3)	61.8 (14.5)	64.4 (17.5)
24	HbA1c response	−13.4 (17.4)	−13.2 (16.7)	−7.7 (13.4)	−10.6 (13.5)	−14.6 (16.7)	−13.3 (17.3)
	Drug name	METFORMIN HYDROCHLORIDE	GLIBENCLAMIDE—20 (0.9)	ALOGLIPTIN—24 (2.3)	PIOGLITAZONE—495 (51.8)	CANAGLIFLOZIN—16 (10.8)	EXENATIDE—46 (32.9)
			GLICLAZIDE—1917 (83.7)	LINAGLIPTIN—115 (11.1)	ROSIGLITAZONE—460 (48.2)	DAPAGLIFLOZIN—124 (83.8)	EXENATIDE MR—14 (10.0)
			GLICLAZIDE MR—121 (5.3)	SAXAGLIPTIN—56 (5.4)		EMPAGLIFLOZIN—8 (5.4)	LIRAGLUTIDE—66 (47.1)
			GLIMEPIRIDE—209 (9.1)	SITAGLIPTIN—793 (76.5)			LIXISENATIDE—14 (10.0)
			GLIPIZIDE −15 (0.7)	VILDAGLIPTIN—49 (4.7)			
			TOLBUTAMIDE—9 (0.4)				
	# Patients (genetic data)	Metformin (*n* = 4162)	SU (*n* = 2060)	DPP4 (*n* = 939)	TZD (*n* = 874)	SGLT2 (*n* = 131)	GLP1 (*n* = 127)

In GoDARTS, the mean age at baseline was 62.3 years, with 58.9% males and 41.1% females. Drug distribution included 47.4% on metformin, 19.8% on sulfonylureas, 14.3% on DPP4 inhibitors, 7.0% on TZDs, 9.7% on SGLT2 inhibitors and 1.8% on GLP‐1 receptor agonists. Full baseline characteristics are shown in Table 1 (further details in the Table S1).

### 3.2 Clinical Variables Associated With Drug Response

For each of the six drug classes, linear regression models were fitted to assess associations between clinical variables and change in HbA1c, defined as the difference between follow‐up and baseline measurements (ΔHbA1c = treatment—baseline). A negative ΔHbA1c indicates a greater reduction in HbA1c (better treatment response), whereas a positive ΔHbA1c reflects a poorer response. Among all classes, DPP‐4 inhibitors demonstrated the smallest mean HbA1c reduction (ΔHbA1c = −7.8 mmol/mol) compared with other drug classes (−11.6 to −16.0 mmol/mol; Table 1).

In the GoDARTS cohort, baseline HbA1c was the variable most strongly associated with glycaemic response across all drug classes (*p* < 0.001), explaining 16%–40% of the total variance in the model (partial *R* ^2^ range 0.16–0.40; Tables S4–S9).

Several clinical variables demonstrated drug class‐specific associations. Shorter diabetes duration was associated with a greater HbA1c reduction with metformin (*p* < 0.001), sulfonylureas (SU) (*p* < 0.001) and TZDs (*p* = 0.03). Higher total cholesterol was consistently associated with poorer glycaemic outcomes across all drug classes (*p* < 0.001) except GLP‐1 receptor agonists. Older age was associated with greater HbA1c reduction in all classes (*p* < 0.001) except SGLT2 inhibitors and GLP‐1 receptor agonists.

Male sex was linked to greater HbA1c reduction with SU (*β* = −1.6 mmol/mol, *p* < 0.001) and SGLT2 inhibitors (*β* = −1.99 mmol/mol, *p* < 0.001), whereas female sex was associated with greater HbA1c reduction with metformin (*β* = −1.78 mmol/mol, *p* < 0.001) and TZD (*β* = −3.2 mmol/mol, *p* < 0.001). Higher BMI was associated with enhanced response to TZDs (*β* = −5.6 mmol/mol, *p* < 0.001), where BMI was log‐transformed in the model, but was associated with poorer response to metformin (*β* = 5.2 mmol/mol, *p* < 0.001) and SU (*β* = 4.5 mmol/mol, *p* < 0.001).

Reduced renal function (higher log‐transformed creatinine) was specifically associated with poorer efficacy of SGLT2 inhibitors (*β* = 3.4 mmol/mol, *p* < 0.001), whereas higher log‐ transformed creatinine was associated with better response to metformin, DPP‐4 inhibitors and TZDs (all *p* < 0.001). Elevated bilirubin was associated with improved response to metformin (*p* < 0.001), SU (*p* < 0.001) and TZD (*p* = 0.01).

In the UK Biobank cohort, the overall pattern of associations was broadly consistent with that observed in GoDARTS, supporting the robustness of the findings (Figure 1; Tables S4–S9). However, some cohort‐specific differences were observed for individual variables. For example, the association between creatinine and SGLT2 inhibitor response differed in direction between the two cohorts, possibly reflecting the smaller number of SGLT2 inhibitor users in UK Biobank and the resulting reduced statistical precision.

**FIGURE 1:**
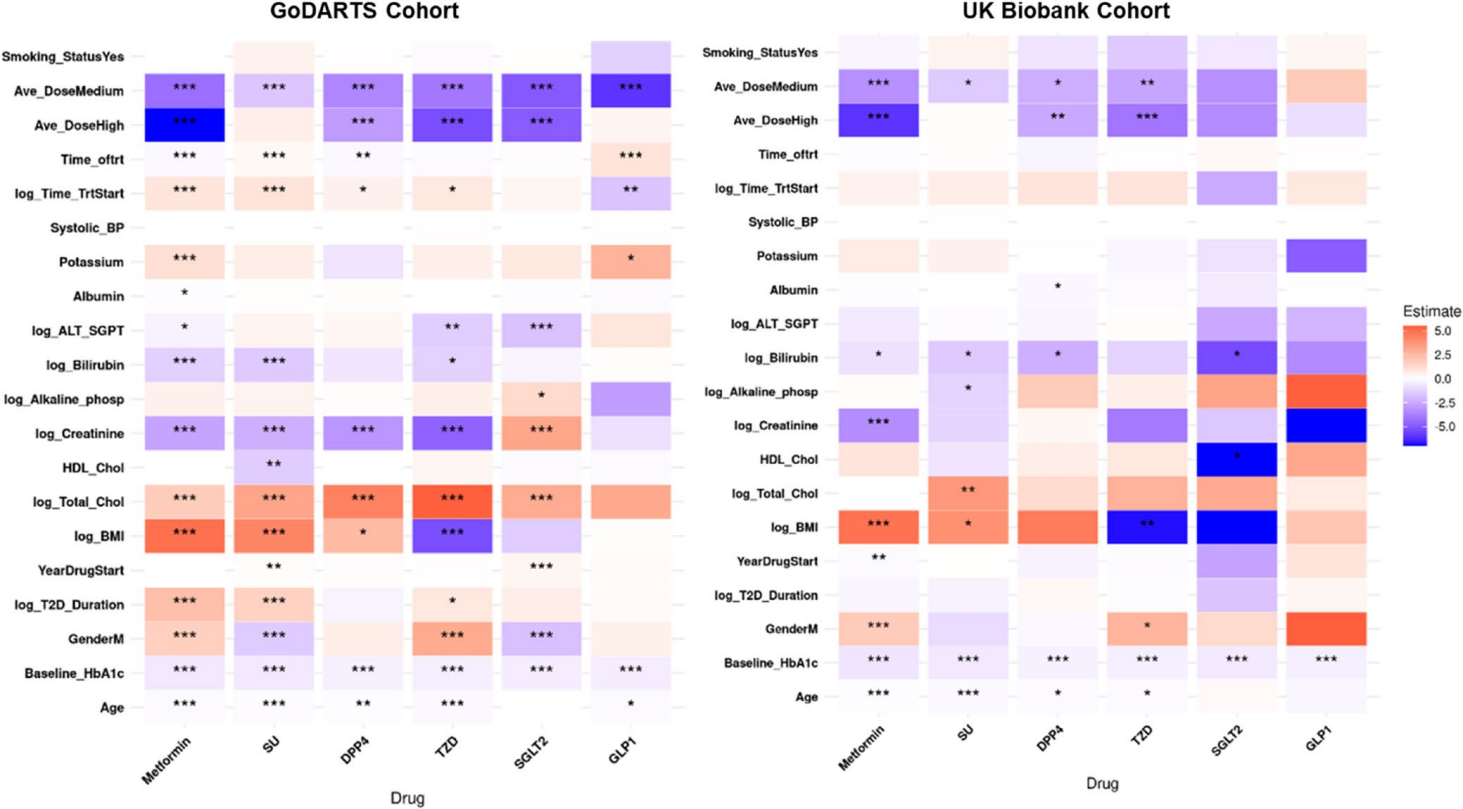
Heatmap showing drug‐class‐specific variables in GoDARTs (left) and UKBiobank (right).

To visualize these relationships, heatmaps for both cohorts are presented in Figure 1, showing drug classes as columns and clinical variables as rows. Red indicates poorer treatment response, whereas blue represents greater HbA1c reduction (better response). All regression coefficients (*β* estimates), *p*‐values and significance details for individual variables are provided in Tables S4–S9.

### 3.3 Genetic Determinants of Drug Response

#### 3.3.1 Overall Genetic Risk Score (GRS) Association With Drug Response

We first examined the association between the total genetic risk score (GRS) and glycaemic response across all six drug classes in both the GoDARTS and UK Biobank cohorts. The GRS was constructed as the aggregate burden of type 2 diabetes (T2D) risk alleles, weighted by their reported effect sizes using variant groupings derived from the Smith et al. [13] soft clustering framework.

Meta‐analysis of results across both cohorts revealed a significant association for sulfonylurea (SU) response: individuals with higher GRS demonstrated greater glycaemic improvement with SU therapy (meta‐analysis *β* = −0.46 mmol/mol, *p* = 0.013). These findings suggest that individuals with a higher genetic burden for T2D experience a stronger glycaemic response to sulfonylureas.

#### 3.3.2 Association of Partitioned‐Polygenic Risk Scores (pPRS) With Drug Response Outcomes

We next evaluated the associations between partitioned polygenic risk scores (pPRS) and ΔHbA1c across drug classes. For each pPRS, three linear regression models were fitted, with β coefficients (mmol/mol per SD increase in pPRS) and corresponding *p*‐values reported in Table S10:

- Model 1: Univariate association between pPRS and ΔHbA1c.
- Model 2: pPRS adjusted for only baseline HbA1c.
- Model 3: Fully adjusted for baseline HbA1c, age, sex, average drug dose, diabetes duration, time to treatment initiation, treatment duration, year of drug start, systolic blood pressure, smoking status and therapy group.

A negative *β* coefficient indicates that a higher pPRS was associated with a greater reduction in HbA1c (better treatment response). A one‐unit change here corresponds to one standard deviation change in risk score.

##### 3.3.2.1 Metformin

In univariate analysis, a higher obesity pPRS was associated with improved response to metformin (meta‐analysis *β* = −0.4 mmol/mol, *p* = 0.02), whereas a higher *β*‐cell 2 pPRS was associated with poorer response (meta‐analysis *β* = 0.53 mmol/mol, *p* = 0.003). In addition, higher VAT.Neg (*p* = 0.02) and proinsulin (*p* = 0.03) pPRS values were associated with poorer glycaemic outcomes.

The *β*‐cell 2 pPRS association remained directionally consistent after adjustment for baseline HbA1c but was no longer statistically significant in the fully adjusted model.

No other pPRS showed significant associations with metformin response in the full model.

##### 3.3.2.2 Sulfonylureas

In univariate analysis, a higher bilirubin pPRS was associated with better sulfonylurea response (meta‐analysis *β* = −0.48 mmol/mol, *p* = 0.04), with the association strengthening after adjustment for baseline HbA1c and clinical covariates (*β* = −0.46 mmol/mol, *p* = 0.01).

Additionally, several β‐cell function related pPRS showed significant associations with sulfonylurea response in the fully adjusted model:

Proinsulin pPRS: *β* = −0.58 mmol/mol, *p* = 0.002.

*β*‐cell 1 pPRS: *β* = −0.44 mmol/mol, *p* = 0.01.

*β*‐cell 2 pPRS: *β* = −0.57 mmol/mol, *p* = 0.002.

##### 3.3.2.3 Thiazolidinediones (TZD)

The ALP‐negative pPRS cluster was associated with poorer glycaemic response to TZD therapy (meta‐analysis *β* = 0.62 mmol/mol, *p* = 0.02), indicating that individuals carrying a higher genetic risk within this pathway experienced reduced HbA1c lowering. A higher hyper‐insulin pPRS was also associated with poorer TZD response (meta‐analysis *β* = 0.59 mmol/mol, *p* = 0.03).

##### 3.3.2.4 DPP‐4 Inhibitors

In univariate analysis, a higher bilirubin pPRS was associated with improved response to DPP‐4 inhibitors (meta‐analysis *β* = −0.66 mmol/mol, *p* = 0.01), with the association strengthening after adjustment for baseline HbA1c and additional clinical covariates (Model 3 *β* = −0.69 mmol/mol, *p* = 0.006). Conversely, a higher ASAT‐positive pPRS was associated with poorer glycaemic outcomes across all three models (meta‐analysis *β* = 0.69 mmol/mol, *p* = 0.006).

##### 3.3.2.5 SGLT2 Inhibitors

A higher liver‐lipid pPRS was associated with improved glycaemic response to SGLT2 inhibitors (meta‐analysis *β* = −0.84 mmol/mol, *p* = 0.05). In contrast, a higher β‐cell 2 pPRS was strongly associated with poorer response in both univariate and adjusted models (meta‐analysis *β* = 1.16 mmol/mol, *p* = 0.009). A higher proinsulin pPRS was also linked to poorer outcomes in univariate and baseline‐adjusted models (*p* = 0.03), though this association did not persist after full adjustment.

##### 3.3.2.6 GLP‐1 Receptor Agonists (GLP‐1RA)

A higher obesity‐related pPRS was associated with better glycaemic response to GLP‐1 receptor agonists in the fully adjusted model (meta‐analysis *β* = −1.49 mmol/mol, *p* = 0.04). Additionally, a higher cholesterol pPRS was associated with poorer response in univariate analysis (*p* = 0.04). Although the effect sizes were modest, the associations remained statistically significant.

Comprehensive results, including β estimates and nominal *p*‐values for all pPRS across drug classes and models, are provided in Table S10. FDR‐adjusted q‐values for the fully adjusted meta‐analysis (Model 3) are also provided in Table S10. Following Benjamini‐Hochberg False Discovery Rate correction, associations involving bilirubin, *β*‐cell 1, *β*‐cell 2, proinsulin and total GRS remained statistically significant for sulfonylurea response, while bilirubin and ASAT‐positive pPRS remained statistically significant for DPP‐4 inhibitor response. The remaining nominal associations did not remain significant after correction and should therefore be interpreted as exploratory.

These findings are visually summarized in Figure 2, which presents forest plots illustrating the association of each pPRS with glycaemic response across all six drug classes. Each plot displays pPRS on the *y*‐axis and corresponding *β* estimates (effect sizes) on the *x*‐axis, with red bars representing UK Biobank results, blue bars representing GoDARTS results and black bars showing the combined meta‐analysis estimates.

**FIGURE 2:**
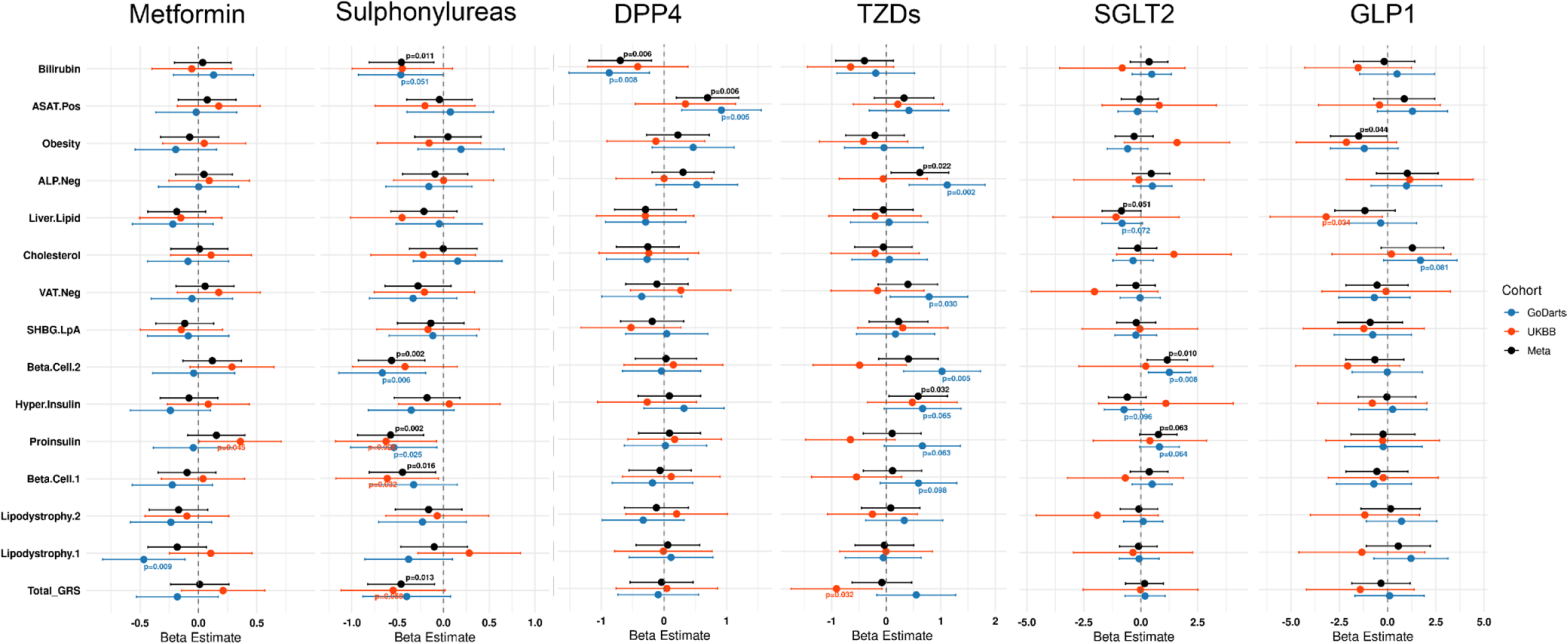
Associations between partitioned polygenic risk scores (pPRS) and glycemic response across drug classes. Each pPRS was analysed in a separate multivariable linear regression model, adjusted for baseline HbA1c, age, sex, average drug dose, diabetes duration, time to treatment initiation, treatment duration, year of drug initiation, systolic blood pressure, smoking status and therapy group. Each estimate corresponds to a separate model including one pPRS at a time. The forest plot displays effect estimates (*β* coefficients and 95% confidence intervals) for all 14 pPRS and total GRS, summarizing their independent associations with change in HbA1c at 12 months post‐treatment initiation. Although all models included the same covariate set, each estimate corresponds to a distinct model including one pPRS at a time.

## 4 Discussion

### 4.1 Key Findings

Our combined analyses show that both routinely measured clinical characteristics and inherited genetic variation contribute to heterogeneity in glycaemic response to glucose‐lowering therapies in type 2 diabetes, although their relative impact differs substantially.

Clinical variables emerged as the dominant determinants of treatment response across all drug classes. In particular, baseline HbA1c consistently explained the largest proportion of variability in glycaemic outcomes, with additional contributions from age and lipid measures that were broadly consistent across therapies. In contrast, factors such as BMI, renal function, sex and diabetes duration showed drug‐specific associations, likely reflecting differences in mechanisms of action between therapies. The consistency of these findings across independent cohorts supports their robustness and reinforces the central role of routinely collected clinical data in guiding treatment decisions.

In parallel, our genetic analyses show that polygenic risk contributes to treatment response in a drug‐class‐specific and biologically meaningful manner. At a global level, higher genetic liability to type 2 diabetes was associated with improved response to sulfonylureas, replicating previous findings reported by Li et al. [27] and supporting the hypothesis that individuals with greater β‐cell dysfunction benefit more from insulin secretagogues [28].

When genetic risk was partitioned into biologically informed pathways, clearer mechanistic relationships emerged: β‐cell related genetic variation was associated with sulfonylurea response, liver‐lipid pathways with SGLT2 inhibitor response and obesity‐related pathways with GLP‐1 receptor agonist response. After correction for multiple testing, associations involving the overall genetic risk score together with bilirubin and β‐cell‐related polygenic risk scores remained statistically significant for sulfonylurea response, while bilirubin and ASAT‐positive polygenic risk scores remained significantly associated with DPP‐4 inhibitor response. No other associations remained statistically significant after correction. The associations that remained significant were consistent across cohorts and broadly aligned with established pharmacological mechanisms.

Despite these biologically coherent associations, the magnitude of genetic effects was modest compared to clinical predictors. As such, while polygenic and pathway‐partitioned scores provide valuable insight into disease mechanisms and drug action, their current utility for individual‐level prediction or clinical stratification remains limited. Instead, their primary contribution lies in enhancing mechanistic understanding and highlighting potential avenues for more precise therapeutic targeting in future studies.

### 4.2 Integration of Clinical Characteristics and Polygenic Risk Scores

To better understand how different sources of variation contribute to treatment response, we evaluated clinical and genetic factors in parallel rather than within a single unified model. This approach was chosen to preserve interpretability and to reduce the risk of collinearity between closely related variables, such as BMI and obesity‐related polygenic scores, which could otherwise obscure true associations.

By analysing these domains separately, we were able to more clearly distinguish their respective roles. Clinical variables primarily capture observable phenotypic variation, while pathway‐partitioned polygenic scores provide insight into underlying biological mechanisms that are not directly measurable in routine care. This complementary framework allowed us to map treatment heterogeneity onto both clinical presentation and genetic architecture without over‐adjustment.

Importantly, the use of biologically informed genetic clustering enabled a more nuanced interpretation of drug response, linking variability to specific physiological pathways such as β‐cell function, lipid metabolism and obesity‐related processes. This adds a layer of mechanistic context that is not accessible through clinical variables alone. Overall, this parallel analytical strategy provides a structured way to integrate clinical and genetic evidence conceptually, even in the absence of a combined predictive model.

### 4.3 Alignment With Existing Literature

Previous studies have consistently demonstrated that routinely collected clinical features explain a substantial proportion of inter‐individual variability in response to glucose‐lowering therapies. Early work from the Diabetes Audit and Research in Tayside Scotland (DARTS) and related cohorts showed that baseline HbA1c, age, BMI and diabetes duration are key determinants of response to metformin and sulfonylureas [21, 29, 30]. These findings are closely aligned with the present analysis, where baseline HbA1c emerged as the dominant predictor across all drug classes, with additional clinical variables contributing smaller, therapy‐specific effects.

More recently, Dennis et al. developed the Exeter five‐drug class model to support treatment selection using routinely available clinical data at the time of drug initiation [4]. Their framework focuses on identifying differential responses between therapies to guide optimal drug choice. In contrast, the present study evaluates absolute response within each drug class, allowing a clearer distinction between common and drug‐specific predictors. Earlier work by Dennis et al. also demonstrated that variables such as baseline HbA1c, age, BMI, estimated glomerular filtration rate (eGFR) and alanine aminotransferase can inform pairwise treatment selection, particularly between SGLT2 inhibitors and DPP‐4 inhibitors [31].

A systematic review further reported limited overall evidence for treatment effect heterogeneity for SGLT2 inhibitors and GLP‐1 receptor agonists but identified specific modifiers of response [32]. Reduced renal function was consistently associated with diminished glycaemic response to SGLT2 inhibitors, reflecting their dependence on renal glucose excretion [33], while markers of reduced insulin secretion were linked to poorer response to GLP‐1 receptor agonists.

Baseline HbA1c remained the most consistent predictor of response across drug classes. In addition, observational and trial data have shown that sex and BMI influence differential responses between sulfonylureas and thiazolidinediones, with male sex and lower BMI associated with greater sulfonylurea response, and higher BMI and female sex associated with enhanced response to thiazolidinediones, consistent with their insulin‐sensitising effects [34].

While these studies provide important insights, most have focused on single drug classes or specific treatment comparisons. The present study extends this literature by evaluating clinical predictors across six drug classes within a unified modelling framework and across two independent cohorts, enabling direct comparison of effect sizes and explained variance.

In parallel, pharmacogenomic studies have evolved from single‐variant analyses to polygenic approaches. Early studies identified individual loci, such as ATM and SLC2A2, associated with metformin response, but these explained only a small proportion of variability. Dawed et al. subsequently demonstrated enrichment of genes involved in drug transport and metabolic regulation in relation to metformin response, supporting a pathway‐based understanding of pharmacological effects [35]. Reviews in this field have similarly emphasised the importance of considering the cumulative impact of multiple genetic variants rather than single loci [36].

More recent studies have explored global polygenic risk scores (PRS) as predictors of treatment response. Li et al. [27] showed that higher type 2 diabetes genetic risk was associated with greater glucose and insulin responses to the sulfonylurea glipizide in the SUGAR‐MGH cohort, suggesting that individuals with greater underlying *β*‐cell dysfunction may benefit more from insulin secretagogues.

Evidence for pathway‐partitioned polygenic risk scores is emerging. Srinivasan et al. [37] reported associations between pathway‐specific scores and metformin response in youth‐onset diabetes, while Sheu et al. [38] demonstrated that SNP clusters representing distinct metabolic pathways were associated with differential glycaemic response to metformin monotherapy, particularly for *β*‐cell–related pathways. Furthermore, Hodgson et al. showed that pathway‐partitioned scores capture clinically relevant heterogeneity more effectively than global PRS, with specific clusters associated not only with treatment response but also with disease progression and complications [17].

### 4.4 Impact of Data Availability and Prescribing Era

Data completeness differed across drug classes, reflecting historical changes in prescribing and data recording. Older therapies such as metformin, sulfonylureas and thiazolidinediones were often started during earlier periods when laboratory and prescribing records were less complete, resulting in higher missingness for some biomarkers. Although multiple imputation was used to reduce bias, some uncertainty related to older data cannot be completely ruled out.

In contrast, newer therapies such as SGLT2 inhibitors and GLP‐1 receptor agonists were introduced more recently and were associated with more complete baseline data. However, these drug classes had smaller sample sizes, particularly in UK Biobank, leading to wider confidence intervals, less precise effect estimates and reduced statistical power. Consequently, these findings should be interpreted cautiously and require validation in larger independent cohorts. These contrasting limitations, greater missing data for older drug classes and smaller sample sizes for newer therapies should be considered when comparing effect sizes across treatments.

### 4.5 Strengths and Limitations

This study has several strengths related to both design and data. The large sample size and use of two independent, well‐characterised cohorts, GoDARTS and UK Biobank, enhance the robustness and generalisability of the findings. The availability of detailed clinical and genetic data enabled a comprehensive assessment of treatment response across multiple drug classes within a unified framework. The consistency in both the size and direction of associations across the two cohorts, despite differences in cohort structure, healthcare settings, prescribing practices and population characteristics, strengthens confidence in the results. Replication across independent datasets suggests that the observed findings reflect true biological and clinical patterns rather than cohort‐specific bias.

The use of multivariable linear regression provided a clear and interpretable modelling approach. This enabled direct comparison of predictors across therapies. Reporting β coefficients and partial *R* ^2^ values helped quantify both effect size and the relative importance of each variable. This supported a clearer distinction between shared and drug‐specific effects. Consistent outcome definitions and meta‐analysis across cohorts further strengthened the reliability of the results.

Several limitations should be considered. The modelling approach assumes linear and additive relationships. It may not capture more complex or non‐linear effects. Some unexplained variation may reflect interactions, time‐related changes, treatment adherence, or unmeasured lifestyle and environmental factors. While this approach improves interpretability, more flexible models may capture additional variation.

Although the cohorts were harmonized with respect to phenotype definitions, statistical models and baseline clinical characteristics, formal heterogeneity statistics and random‐effects sensitivity analyses were not performed and may be considered in future validation studies.

The observational design is another limitation. Treatment allocation was not random and may reflect underlying clinical characteristics. This introduces the risk of confounding by indication. Although adjustments were made, residual confounding cannot be ruled out. Use of routine healthcare data also means some variables may be missing or inconsistently recorded. In addition, real‐world treatment patterns, such as variation in adherence, dosing and switching, may influence results and limit comparison with randomised trials.

Differences in genotyping platforms, variant coverage and score construction across studies may introduce variability. However, consistent effect directions across cohorts support the reliability of the findings. Restriction to individuals of European ancestry limits generalisability. The modest genetic effect sizes reflect the complex nature of treatment response. In one clustering framework, missing variants reduced the available sample size. As with all observational genetic studies, causal relationships cannot be confirmed. Further improvements in genetic data, cohort diversity and data harmonization will support future clinical application.

A further consideration is the large number of statistical comparisons performed across pathway‐partitioned polygenic risk scores. To reduce the likelihood of false‐positive findings, False Discovery Rate correction was applied within each drug class. While several associations for sulfonylureas and DPP‐4 inhibitors remained statistically significant after correction, most associations observed for the remaining drug classes did not. Although several additional pathway‐specific associations reached nominal statistical significance, these did not survive FDR correction. These findings should therefore be considered hypothesis‐generating and require replication in larger, independent cohorts before their clinical relevance can be established.

### 4.6 Implications for Personalized Medicine

Pathway‐partitioned polygenic risk scores mainly improve biological understanding of differences in treatment response. Their role in direct clinical decision‐making is currently limited. These scores link variation in drug response to specific physiological pathways, such as β‐cell function, lipid metabolism and obesity‐related processes. This provides mechanistic insight that is not captured by routine clinical measures.

The significant associations observed for sulfonylureas and DPP‐4 inhibitors support the biological relevance of inherited disease pathways in determining treatment response and provide further evidence that pathway‐informed polygenic risk scores may contribute to future precision medicine strategies. However, the remaining genetic associations should be considered exploratory until replicated in independent cohorts.

This is likely to improve as genetic models develop further. Advances in variant discovery, inclusion of rare variants and clearer pathway definitions may increase both accuracy and interpretability. Overall, polygenic approaches represent a step toward more mechanism‐based patient stratification.

### 4.7 Future Directions

Further research is needed to build on these findings. Replication in additional cohorts is important, including both independent European datasets and more diverse populations. Expanding beyond European ancestry will improve generalisability and help reduce potential health inequalities [12, 39, 40].

Future work should also extend beyond genetics. Integration of other biological data types, such as proteomics and metabolomics, may help clarify the mechanisms underlying treatment response [41]. Combining these data with clinical and genetic information could improve both prediction and biological understanding.

Advanced analytical approaches could also be explored. Machine learning and deep learning models, including transformer‐based methods, may help capture patient trajectories over time and improve understanding of treatment response and progression to diabetes‐related complications [42, 43, 44, 45]. Translational efforts are also needed to support clinical application. This includes the development of practical tools and models that can incorporate complex data and provide clear, patient‐level guidance for treatment decisions.

## 5 Conclusion

In conclusion, this study demonstrates that routinely collected clinical characteristics explain a substantial proportion of variability in glycaemic response across multiple glucose‐lowering drug classes, with consistent findings across independent cohorts. At the same time, pathway‐partitioned polygenic risk scores reveal biologically meaningful, drug‐class‐specific associations that provide insight into the mechanisms underlying treatment heterogeneity.

Together, these findings support a complementary framework in which clinical factors define the current foundation for treatment stratification, while genetic information enhances biological understanding and offers potential for future refinement. By establishing a robust and interpretable clinical baseline and extending this with pathway‐informed genetic analyses, this work contributes to the evolving landscape of precision medicine in type 2 diabetes and highlights the importance of integrating clinical and biological data to improve therapeutic decision‐making.

## Author Contributions

E.R.P., R.K. and R.G. contributed to conceptualization and supervision. S.G. conducted the data analysis and wrote the original manuscript. L.D. contributed to data curation and interpretation. S.G., E.R.P., R.K. and R.G. contributed to the reviewing and editing.

## Funding

This work was supported by funding from Novo Nordisk, which provided a PhD studentship to S.G. and University of Dundee.

## Ethics Statement

Data provision and linkage were carried out by the University of Dundee Health Informatics Centre, with analysis of anonymized data performed in an ISO27001 and Scottish Government‐accredited secure safe haven. Health Informatics Centre standard operating procedures were reviewed and approved by the National Health Service (NHS) East of Scotland Research Ethics Service (22/ES/0034), and consent for this study was obtained from the NHS Fife Caldicott Guardian. Under these approvals, secondary analysis of anonymised routine healthcare data does not require additional participant consent or compensation.

## Conflicts of Interest

R.G. is employed by and owns stock in Novo Nordisk and Disease Intelligence Pte Ltd.

## Supporting information


**Table S1:** Definition of input variables used in the regression models.
**Table S2:** Predictors used in clinical and genetic models.
**Table S3:** R packages used in the statistical analysis.
**Table S4:** Summary of linear regression models showing associations between clinical covariates with Metformin response [outcome, e.g., change in HbA1c] in GoDARTs and UKBioBank.
**Table S5:** Summary of linear regression models showing associations between clinical covariates with Sulphonylurea response [outcome, e.g., change in HbA1c] in GoDARTs and UKBioBank.
**Table S6:** Summary of linear regression models showing associations between clinical covariates with Thiazolidinediones (TZD) response [outcome, e.g., change in HbA1c] in GoDARTs and UKBioBank.
**Table S7:** Summary of linear regression models showing associations between clinical covariates with DPP4 response [outcome, e.g., change in HbA1c] in GoDARTs and UKBioBank.
**Table S8:** Summary of linear regression models showing associations between clinical covariates with SGLT2 response [outcome, e.g., change in HbA1c] in GoDARTs and UKBioBank.
**Table S9:** Summary of linear regression models showing associations between clinical covariates with GLP1 response [outcome, e.g., change in HbA1c] in GoDARTs and UKBioBank.
**Table S10:** Association of Partitioned Polygenic Risk Scores (pPRS) with drug response outcomes.

## Data Availability

Data provision and linkage were carried out by the University of Dundee Health Informatics Centre, with analysis of anonymized data performed in an ISO27001 and Scottish Government–accredited secure safe haven. Health Informatics Centre standard operating procedures were reviewed and approved by the National Health Service (NHS) East of Scotland Research Ethics Service (22/ES/0034), and consent for this study was obtained from the NHS Fife Caldicott Guardian. Under these approvals, secondary analysis of anonymised routine healthcare data does not require additional participant consent or compensation.
